# Supporting the decision for female genital mutilation/cutting and its predictors among healthcare providers in Upper Egypt

**DOI:** 10.1186/s12913-026-14028-w

**Published:** 2026-02-04

**Authors:** Doaa Mohamed Mahmoud Osman, Nina Van Eekert, Heba Mahmoud Mohammed

**Affiliations:** 1 Public Health and Community Medicine Department, Faculty of Medicine, Assiut University, Asyut City, Egypt; 2 The Research Foundation – Flanders (12g3322N), Brussels, Belgium

**Keywords:** Egypt, Female genital mutilation/cutting, Healthcare providers, Medicalization

## Abstract

**Background:**

In Egypt, a growing number of female genital mutilation/cutting (FGM/C) procedures among girls are conducted by professional healthcare providers (HCPs). This study aimed to identify the prevalence of health professionals’ support for FGM/C and its predictors among HCPs in Upper Egypt.

**Methods:**

A cross-sectional study was conducted among 385 HCPs working in the gynaecology and obstetrics specialty for the Egyptian Ministry of Health in the Asyut Governorate. Data was collected using a self-administered questionnaire that assessed personal characteristics, HCPs training in and knowledge of FGM/C, the HCPs’ attitudes towards the practice of FGM/C, as well as their involvement in the practice (medicalized FGM/C), FGM/C in their own family and the decision to support FGM/C for girls when consulted.

**Result:**

Almost one third of the HCPs studied supported the decision for FGM/C or were not clearly against FGM/C (34.3%) for girls when consulted. The adjusted predictors of support for FGM/C or not being clearly against FGM/C among Egyptian HCPs were: increasing age (AOR = 1.03, 95% CI: 1.01–1.06), low scores on attitude against medicalized FGM/C (AOR = 0.610, 95% CI: 0.49–0.75) and the predominance of FGM/C in the area where HCPs were raised (AOR = 2.410, 95% CI: 1.22–4.75).

**Conclusions:**

There is still a substantial proportion of Egyptian HCPs who support FGM/C for girls or are not clearly against FGM/C when consulted. There is a need to intensify efforts to direct education programmes towards HCPs, especially those who are older, who have a positive attitude towards FGM/C medicalization and who were raised in areas with a predominance of FGM/C.

**Supplementary Information:**

The online version contains supplementary material available at 10.1186/s12913-026-14028-w.

## Introduction

Female genital mutilation/cutting (FGM/C) is a surgical procedure that is not recommended in medical textbooks [1]. It is a harmful practice with no health benefits, which involves removing and injuring healthy and normal female genital tissue, interfering with the natural functions of girls’ and women’s bodies [2].

The World Health Organization (WHO) defines FGM/C as all procedures involving partial or total removal of the female external genitalia or other injury to the female genital organs for non-medical reasons [3]. FGM/C is considered a violation of the fundamental human rights of girls and women, and its abandonment is integrated into the 5.3 sustainable development goal of ‘Gender Equality’. It reflects an aspect of deep-rooted inequality between the genders and represents an extreme form of discrimination against girls and women [4–6].

FGM/C is a global public health concern. It is commonly practised in the western, eastern and northeastern regions of Africa and some countries in the Middle East and Asia [4]. Moreover, FGM/C has been reported in Australia, Europe and North America in migrant communities originating from countries in which FGM/C is practised [7]. According to data available from WHO, across 30 countries, there are more than 200 million girls and women alive today who have been subjected to FGM/C. Annually, more than 3 million girls would be at risk of FGM/C [4].

FGM/C is a complex and culturally shaped phenomenon [8]. Higher levels of FGM/C are correlated with some sociodemographic and cultural factors, including: low socioeconomic standards; illiteracy; residence in rural areas; girls whose mothers underwent FGM/C; responding to community pressure to conform to local and long-established norms to evade stigmatization and to gain social acceptance; false beliefs that FGM/C is required by religious rules and that it is beneficial to women as it improves hygiene and prevents infidelity [9, 10].

All forms of FGM/C are associated with increased immediate health risks, including death, severe pain, excessive haemorrhaging, infections and psychological and psychiatric problems. It also has a variety of long-term complications, such as menstrual problems, sexual problems and obstetric complications [2, 11].

Parents have now turned to healthcare providers (HCPs) for the informal medical performance of FGM/C believing that this will mitigate and protect their girls from FGM/C complications [1]. This has led to one of the major shifts in FGM/C today – its medicalization. This medicalization of Female Genital Mutilation/Cutting (mFGM/C) refers to conditions in which FGM/C is practised by any category of healthcare provider, whether in a public or a private clinic, at home or elsewhere. The medicalization of FGM/C also involves exposure to reinfibulation procedures at any point in time in a woman’s life [12].

FGM/C is unacceptable from the perspective of human rights and public health, regardless of who performs it. WHO is opposed to the medicalization of all forms of FGM/C and strongly urges HCPs not to carry out FGM/C, even when their patient or their patient’s family requests it. These arguments are based on the assumption that the medicalization of FGM/C impedes efforts to abandon the practice and results in a setback to global efforts to eradicate this harmful practice [7]. Moreover, its false legitimization by the medical field may give the green light to its performance by non-healthcare specialists and a consequent rise in the incidence of complications [13].

The practice of FGM/C is prevalent in Egypt. Data from the Egyptian Family Health Survey (EFHS) in 2021 revealed that 85.6% of women aged 15–49 had undergone FGM/C; 14.2% of daughters aged 0–19 years had undergone FGM/C; and 27% of girls were expected to undergo FGM/C. In 2021, Upper Egypt had the highest percentages of FGM/C among girls aged less than 19 years (23.7%) and almost 44% of girls were expected to undergo FGM/C [14].

Furthermore, the percentage of medicalized procedures has significantly increased across different birth cohorts. For women born between 1960 and 1969, only 14% of FGM/C procedures were performed by a health professional. However, this percentage has steadily risen over the years, reaching 83% for the youngest cohort of women born between 2000 and 2009 [15].

Egypt’s Constitution (2014) states that children should be provided with care and protection from all forms of violence, abuse, mistreatment and commercial and sexual exploitation. It also states that women should be protected against all forms of violence and be enabled to strike a balance between family duties and work requirements [16].

Moreover, female genital mutilation has been criminalized in Egypt, whether undertaken by non-medical or medical personnel [17]. However, the current contribution of HCPs to FGM/C is still evident in Egypt, where almost 75% of FGM/C among girls aged 0–19 (FHS, 2021, 73.8%) was performed by a doctor, nurse or other healthcare worker [14].

What remains largely unaddressed in the debate about the shift towards the medicalization of FGM/C is an understanding of this medicalization through the optics of HCPs themselves. Previous research has mapped the main reasons for HCPs performing FGM/C, such as being a source of financial gain, conforming to social norms and expectations, and believing falsely that medicalization reduces the harm of the practice [18]. However, it remains unclear how widespread professional HCP support for FGM/C is and when this support translates into actual engagement in practice. Thus, the aim of the current study was to identify the prevalence of HCPs’ support for FGM/C and its predictors among physicians and nurses working in the gynaecology and obstetrics specialty in Asyut Governorate, Upper Egypt.

## Methods/study design

### Study design and the study population

A cross-sectional study was conducted in Asyut Governorate, which is one of the largest governorates in Upper Egypt, with approximately 5,061,934 inhabitants in 2024, according to the Central Agency for Public Mobilization and Statistics (CAPMAS). The study included 385 HCPs (both physicians and nurses) who work in the specialty of gynaecology and obstetrics in this governorate.

The HCPs studied were recruited from different hospitals of the Egyptian Ministry of Health and Population (Asyut General Hospital, El Eman General Hospital, El Eman Gynaecology and Obstetrics Hospital in Asyut City and other district hospitals in Asyut Governorate). Data was collected from January to June 2022.

### Sample size and technique

A purposive non-probability sampling technique was applied. The HCPs who participated in the survey were recruited from their work site or during their attendance at scientific meetings for gynaecology and obstetrics. The researchers collected data from all the nursing staff and some physicians in the hospital. The data from the remaining physicians was collected during their attendance of a scientific meeting held to train them and increase their scientific knowledge.

The sample size was estimated using the EPI info statistical package Version 7.2.01. The parameters used to estimate the sample size for a cross-sectional study design included 50% as a hypothesized proportion of those who would support the decision for FGM/C (due to the absence of a similar previous study), a confidence level of 95% and a margin of error of 5%. The minimum estimated sample size was 384 HCPs.

### Data collection

Data was collected using an anonymous self-administered questionnaire. The researchers designed and developed the questionnaire in English and then translated the questionnaire into Arabic. A backward translation of the questionnaire was performed by two gynaecology and obstetrics specialists who were not familiar with the original English questionnaire. Revision of the translated questionnaire was done by a linguistic professional and two professors from two different specialties (public health and gynaecology and obstetrics). The questionnaire was distributed among qualified Egyptian HCPs in their native Arabic to ease their comprehension and completion of the questionnaire. An English version of the questionnaire can be found as Supplementary File 1. The questionnaire consisted of eight main domains that covered:

1. Personal characteristics: age, sex, birth place, current residence, educational level (Bachelor’s, Master’s, PhD or MD) and professional specialty [19].
2. Training in FGM/C: whether HCPs had previously heard about FGM/C during their undergraduate education or received training in FGM/C after employment [20].
3. Knowledge about FGM/C: this included their definition of FGM/C; their understanding of the prevalence of FGM/C among girls in Egypt; their description of types of FGM/C; whether they thought FGM/C has negative consequences for women’s health; whether they would report these consequences, if yes; and their awareness of the existence of an Egyptian law that criminalizes the professional practice of FGM/C [20–23].
4. Healthcare providers’ general attitude towards FGM/C: this included 13 statements on FGM/C to which each HCP answered whether they agreed, were neutral or disagreed. The statements were: (i) FGM/C is a mandatory religious practice; (ii) FGM/C reduces sexual feeling; (iii) FGM/C is a rite of passage for girls transitioning into womanhood; (iv) FGM/C reduces prostitution; (v) FGM/C maintains a girl’s virginity; (vi) FGM/C is a deeply rooted, good cultural practice; (vii) FGM/C is considered a good practice; (viii) FGM/C is a hygienic practice for girls; (ix) FGM/C is necessary for women to be marriageable; (x) FGM/C does not violate human rights; xi) Girls who do not undergo FGM/C should be discriminated against; xii) No form of FGM/C should be allowed; and xiii) FGM/C preserves the cosmetic appearance of girls genitalia [23, 24].

Each participant’s total attitude score was calculated based on their responses: ‘disagree’ scored 3; ‘neutral’ scored 2; and ‘agree’ scored 1. The scoring was reversed for the statement: ‘No form of FGM/C should be allowed’. A higher attitude score indicated a more negative attitude of the HCP towards the practice of FGM/C in the community.

5. HCPs’ attitudes towards the professional practice of FGM/C (medicalized FGM/C): HCPs were requested to respond to the following six statements with either yes or no: (i) It is necessary to continue FGM/C in Egypt; (ii) It is possible to eliminate FGM/C in Egypt; (iii) HCPs have a role in eradicating this practice; (iv) The medicalization of FGM/C would make it a safe practice; (v) The medicalization promote the practice in the community; and (vi) The practice should be stopped at all levels [23, 24].

 Each participant’s total attitude score was calculated based on their responses. Each positive response against the medicalization of FGM/C was given two points, while responses that supported the professional practice of FGM/C were given one point. The higher the score the more negative the attitude of the HCP towards the professional practice of FGM/C and thus its medicalization.

6. HCPs were also asked about FGM/C in their families and their intention to have their girls undergo FGM/C in the future.
7. HCPs’ experience with FGM/C in clinical practice: the HCPs were asked whether they had previously examined a girl who underwent FGM/C or had seen a girl who presented with FGM/C complications in their work. They were also asked whether they had ever carried out FGM/C [23, 25].
8. FGM/C consultations: The authors presented the HCPs with a scenario of a woman coming to see them with her daughter or another female relative and requesting the HCP to perform FGM/C on the girl. The HCP answer options were: s/he would perform FGM/C; s/he would refer girl to another physician to perform the practice; s/he would examine the girl first then decide if it was needed; s/he would educate the mother encouraging her to not have FGM/C performed on her daughter; s/he would not perform the practice; or s/he would not perform the practice and would also educate the mother.

### Data management and analysis

Data analyses were performed using the Statistical Package for Social Science (SPSS), version 26.0 for Windows. Quantitative data was described in the form of means and standard deviations, while frequencies and percentages were used to present categorical data. The scales used were tested for reliability and internal consistency. Cronbach’s alpha values were acceptable for the general attitude towards FGM/C scale (0.837) and for the scale of attitude towards professional practice of FGM/C (0.699).

Supporting the decision for FGM/C or not clearly being against FGM/C was considered as the outcome variable. This was created after recoding the response of each HCP to the scenario of a woman coming with her daughter or relative and asking the HCP to perform FGM/C on the girl. HCPs who stated that they would perform FGM/C; would examine the girl to determine the need for FGM/C; or would refer the case to other physicians to perform FGM/C were classified into the category of supporting FGM/C or not being clearly against the practice. HCPs who stated that they would refuse to carry out FGM/C; would educate the mothers; or would do both were classified into the category of not supporting FGM/C. There is no scientific medical evidence that supports the harmful practice of FGM/C. It is a violation of the rights of the child and an illegal practice that is prohibited in Egyptian law. The authors thus considered that the only responses of the HCPs that were clearly *not* in support of FGM/C were refusal to perform FGM/C and/or educating the mother by explaining that it is an illegal and unethical practice that violates the human rights of the child. The authors thus considered all of the remaining responses of the HCPs – performing FGM/C, examining girls to determine the need for FGM/C, or referring the case to other physicians to perform FGM/C – to be supportive of FGM/C or not clearly against FGM/C.

For bivariate analysis, the Chi-square test was applied to analyse the difference in categorical variables, while a student sample T-test was used to compare the mean values between two independent groups. An adjusted multivariate logistic regression model was applied to identify predictors of supporting FGM/C or not being clearly against FGM/C as an outcome variable (supporting FGM/C or not clearly against FGM = 1 and not supporting FGM/C = 0). The explanatory variables entered into the adjusted regression models were: age and sex (priori variables) and the significant variables that were the result of the bivariate analysis. The significance of statistical tests was considered if the P-value was less than 0.05. Microsoft Excel 2016 was used to enter data and construct graphic presentations.

## Results

Table 1 presents the personal characteristics of the HCPs involved in the study. The mean HCP age was 37.30 ± 9.99 and ranged from 20 to 76 years. Females made up approximately three quarters (77.4%) of the total number of HCPs in the study and also made up the majority of nurses (98.4%). Both the original and current residences of the respondents were primarily located in urban areas. The majority of nurses held a diploma in nursing (78.7%), while a large proportion of physicians (62%) had a Master’s or MD degree. About half of the HCPs reported studying FGM/C in their undergraduate curriculum, while only one third (30.4%) reported having previous training in FGM/C.

**Table 1:** Characteristics of the HCPs studied, upper Egypt

Variable	Total (*n* = 385)	Physicians (*n* = 197)	Nurses (*n* = 188)
**Age**			
• Mean ± SD	37.30 ± 9.99 (20–76)	37.34 ± 10.46	37.25 ± 9.49
**Sex**			
• Male	87 (22.6%)	84 (42.6%)	3 (1.6%)
• Female	298 (77.4%)	113 (57.4%)	185 (98.4%)
**Original residence**			
• Urban	259 (67.3%)	147 (74.6%)	112 (59.6%)
• Rural	126 (32.7%)	50 (25.4%)	76 (40.4%)
**Current residence**			
• Urban	310 (80.5%)	179 (90.9%)	131 (69.7%)
• Rural	75 (19.5%)	18 (9.1%)	57 (30.3%)
**Qualification**			
• Bachelor’s degree	105 (27.3%)	75 (38.1%)	30 (16.0%)
• Diploma in nursing/Nursing institute graduate	148 (38.4%)	-------------	148 (78.7%)
• Master’s degree	119 (30.9%)	113 (57.4%)	6 (3.2%)
• Doctoral degree	13 (3.4%)	9 (4.6%)	4 (2.1%)
**Job site**			
• University Hospital	163 (42.3%)	60 (30.5%)	103 (54.8%)
• Ministry of Health	222 (57.7%)	137 (69.5%)	85 (45.2%)
**Previously studied FGM/C in curriculum**			
• Yes	195 (50.6%)	106 (53.8%)	89 (47.3%)
• No	151 (39.2%)	72 (36.5%)	79 (42.0%)
• Do not remember	39 (10.1%)	19 (9.6%)	20 (10.6%)
**Previously received training about FGM/C**			
• Yes	117 (30.4%)	72 (36.5%)	45 (23.9%)
• No	259 (67.3%)	120 (60.9%)	139 (73.9%)
• Do not remember	9 (2.3%)	5 (2.5%)	4 (2.1%)

Table 2 displays the knowledge of the HCPs about FGM/C, their personal experiences, their general attitude towards FGM/C and their attitude towards the professional practice of FGM/C (medicalized FGM/C). The majority (97%) correctly defined FGM/C, while only one fifth of participants (21.3%) identified the prevalence of FGM/C in Egypt. A large proportion (85%) of HCPs reported that FGM/C has negative consequences. The most commonly reported negative effects, in order, were: psychological, reduction of sexual orgasm, bleeding, infection transmission and dyspareunia. Approximately 71% of the HCPs were aware of the existence of a law prohibiting HCPs from carrying out FGM/C. The proportion of HCPs aware of the law was significantly higher among physicians (80.7%) than among nurses (60.1%). Nurses reported significantly higher levels of FGM/C prevalence in their families and the areas they grew up in compared to physicians.

**Table 2:** Knowledge about FGM/C, personal experience and attitude towards FGM/C and its medicalization among the HCPs studied, upper Egypt

Variable	Total (*n* = 385)	Physicians (*n* = 197)	Nurses (*n* = 188)	*P*-Value*
**Correctly defined FGM/C**				
• Yes	373 (96.9%)	191 (97.0%)	182 (96.8%)	0.934
• No/Don’t know	12 (3.1%)	6 (3.0%)	6 (3.2%)	
**Correctly identified the prevalence of FGM/C in Egypt**				
• Correct answer	82 (21.3%)	44 (22.3%)	38 (20.2%)	0.305
• Wrong answer	13 (3.4%)	4 (2.0%)	9 (4.8%)	
• Do not know	290 (75.3%)	149 (75.6%)	141 (75.0%)	
**FGM/C has negative consequences for women’s health**				
• Yes	326 (84.7%)	169 (85.8%)	157 (83.5%)	0.104
• No	25 (6.5%)	8 (4.1%)	17 (9.0%)	
• Do not know	34 (8.8%)	20 (10.2%)	14 (7.4%)	
**Negative health consequences**	*N* = 326	*N* = 169	*N* = 157	
• Psychological	262 (80.4%)	149 (88.2%)	113 (72.0%)	**< 0.001**
• Transmission of infectious diseases	136 (41.7%)	81 (47.9%)	55 (35.0%)	**0.018**
• Bleeding	211 (64.7%)	123 (72.8%)	88 (56.1%)	**0.002**
• Scar formation	134 (41.1%)	92 (54.4%)	42 (26.8%)	**< 0.001**
• Difficulty during pregnancy	63 (19.3%)	38 (22.5%)	25 (15.9%)	0.134
• Difficulty during delivery	91 (27.9%)	62 (36.7%)	29 (18.5%)	**< 0.001**
• Reduction of sexual orgasm	237 (72.7%)	130 (76.9%)	107 (68.2%)	0.076
• Pain during sexual relation	131 (40.2%)	87 (51.5%)	44 (28.0%)	**< 0.001**
**Is there is a law prohibiting FGM/C in Egypt**				
• Yes	272 (70.6%)	159 (80.7%)	113 (60.1%)	**< 0.001**
• No	113 (29.4%)	38 (19.3%)	75 (39.9%)	
**FGM/C is common in areas where HCP grew up**				
• Yes	255 (66.2%)	110 (55.8%)	145 (77.1%)	**< 0.001**
• No	65 (16.9%)	48 (24.4%)	17 (9.0%)	
• Do not know	65 (16.9%)	39 (19.8%)	26 (13.8%)	
**FGM/C is common in HCP family/household**				
• Yes	214 (55.6%)	70 (35.5%)	144 (76.6%)	**< 0.001**
• No	171 (44.4%)	127 (64.5%)	44 (23.4%)	
**Ever carried out FGM/C**				
• Yes	51 (13.2%)	19 (9.6%)	32 (17.0%)	**0.033**
• No	334 (86.8%)	178 (90.4%)	156 (83.0%)	
**Previously examined a girl who underwent FGM/C**				
• Yes	342 (88.8%)	186 (94.4%)	156 (83.0%)	**< 0.001**
• No	43 (11.2%)	11 (5.6%)	32 (17.0%)	
**Previously saw a girl with FGM/C complications**				
• Yes	254 (66.0%)	142 (72.1%)	112 (59.6%)	**0.010**
• No	131 (34.0%)	55 (27.9%)	76 (40.4%)	
**Intention to have FGM/C performed on their future daughter**				
• Yes	43 (11.2%)	8 (4.1%)	35 (18.6%)	**< 0.001**
• No	279 (72.5%)	164 (83.2%)	115 (61.2%)	
• Do not know	63 (16.4%)	25 (12.7%)	38 (20.2%)	
**General attitude towards FGM/C**	32.96 ± 5.42	34.51 ± 4.18	31.34 ± 6.06	**< 0.001**
**Attitude towards professional practice/medicalization of FGM/C**	10.21 ± 1.67	10.52 ± 1.45	9.89 ± 1.82	**< 0.001**

In their clinical practice, a majority of HCPs reported having examined a girl who underwent FGM/C (88.7%). Additionally, two thirds of the HCPs (66.0%) reported encountering cases involving complications related to FGM/C. These percentages were higher among physicians, with 94.4% having examined a girl who underwent FGM/C and 72.2% reporting cases of FGM/C complications, while for nurses, the percentages were 83% and 59.6%, respectively. Looking at HCPs actually carrying out FGM/C themselves, we see that a larger share of nurses had carried out FGM/C (17.0%), compared to physicians (9.6%). In total, this was equivalent to 13.2% of the HCPs. In addition, a larger share of nurses had the intention to perform FGM/C on their own future daughter (18.6%), compared to 4.1% in the physicians.

The mean of the HCPs’ general attitude towards FGM/C was 32.96 ± 5.42, while the mean of their attitude towards the professional practice of FGM/C and thus its medicalization was 10.22 ± 1.67. Physicians had a more negative attitude towards FGM/C than nurses, with the physicians having significantly higher mean values for attitudes against FGM/C, both in general and in relation to its professional practice, compared to nurses (*P* < 0.001).

**Fig. 1:**
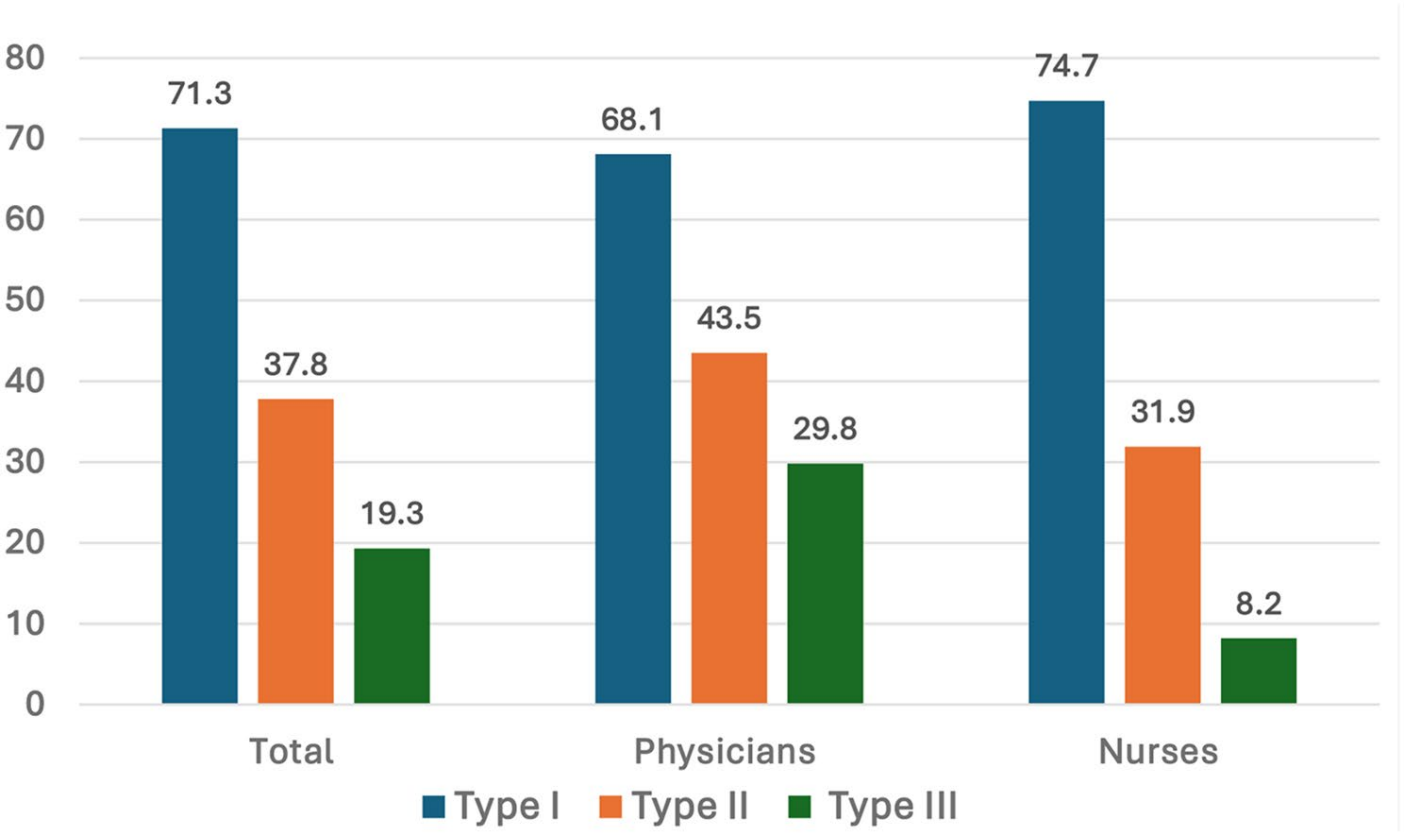
Awareness of FGM/C types among HCPs studied, Upper Egypt. Data presented in percentages

Figure 1 presents the knowledge of the HCPs about types of FGM/C. Type I FGM/C was most commonly identified (71.3%), followed by Type II (37.8%) and Type III (19.3%). None of the HCPs identified Type IV FGM/C. A higher proportion of the physicians were aware of the different FGM/C types, compared to nurses, except Type I.

**Fig. 2:**
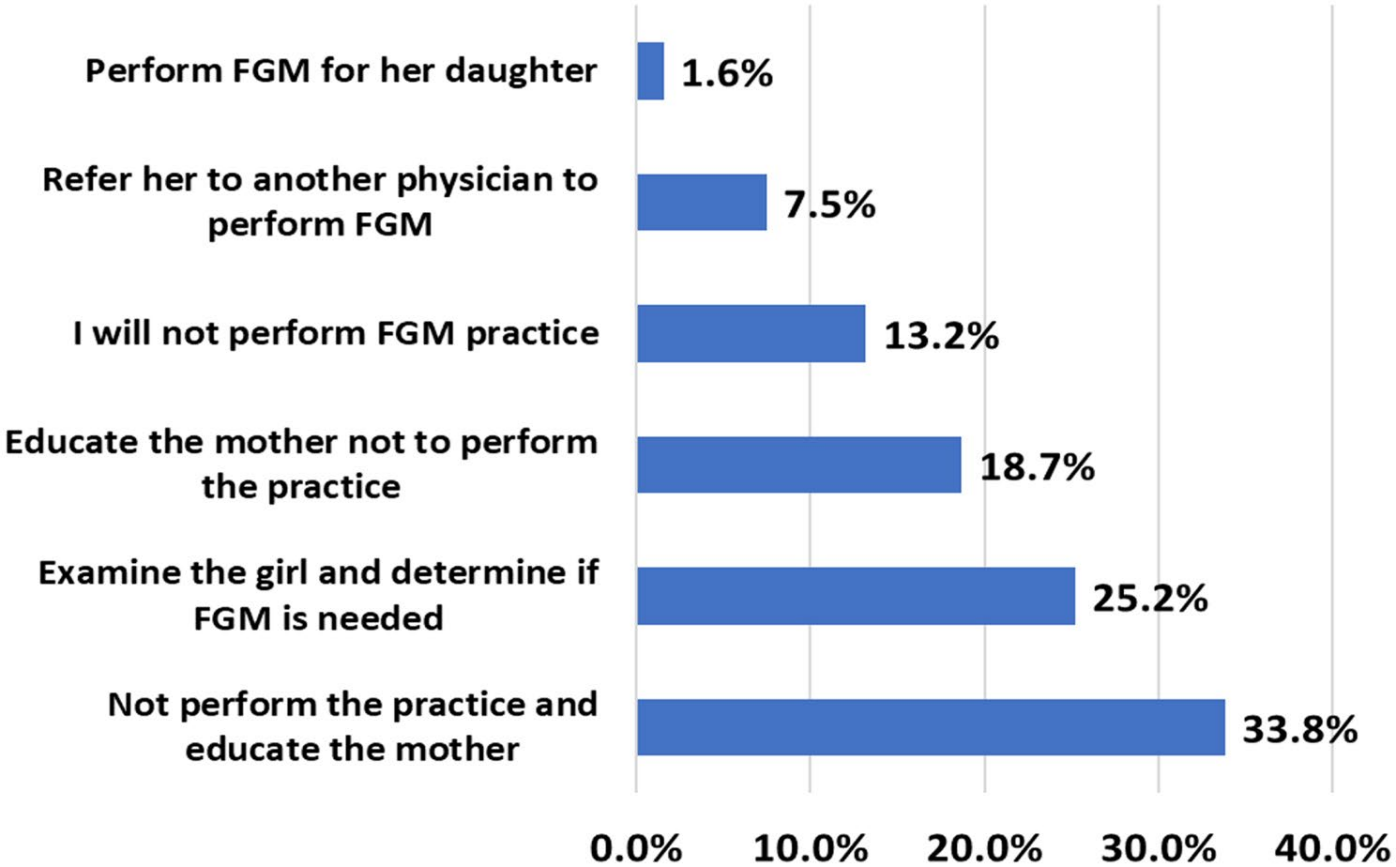
Reponses of HCPs to mothers wishing to have FGM/C performed on their daughters, Upper Egypt. Data presented in percentages

Figure 2 shows the responses of HCPs about support for the professional practice of FGM/C, given to mothers wishing to have FGM/C performed on their daughters. Approximately one third of the HCPs studied supported the professional practice of FGM/C or were not clearly against it (34.3%) in the scenario of being consulted about FGM/C. They either stated that they would examine the girl to determine the need for FGM/C (25.2%); would perform FGM/C themselves (1.6%); or would provide a referral to other physicians to perform FGM/C (7.5%). A large proportion (65.7%) of HCPs did not support FGM/C, stating that they would refuse to perform it, would educate the mothers, or would do both.

Table 3 shows the variables associated with supporting FGM/C or not being clearly against FGM/C among HCPs in the scenario of being consulted by mothers. A significantly higher proportion (37.2%) of female HCPs compared to male HCPs (24.1%) supported the practice or were not clearly against FGM/C (*P* = 0.023). Similarly, a higher proportion of FGM/C support or not being clearly against FGM/C was detected among nurses (42.0%) compared to physicians (26.9%) (*P* = 0.002). Age, job location and both original and current residence were not significantly associated with supporting FGM/C or not being clearly against it (*P* > 0.05).

**Table 3:** Variables associated with HCPs supporting FGM/C as a professional practice, upper Egypt

Variable	Not supporting FGM/C as professional practice (*n* = 253)	Supporting FGM/C as professional practice/ not clearly against FGM (*n* = 132)	*P*-Value*
**Age**	36.75 ± 9.59	38.35 ± 10.65	0.136
**Sex**			
• Male	66 (75.9%)	21 (24.1%)	**0.023**
• Female	187 (62.8%)	111 (37.2%)	
**Original residence**			
• Urban	175 (67.6%)	84 (32.4%)	0.272
• Rural	78 (61.9%)	48 (38.1%)	
**Current residence**			
• Urban	207 (66.8%)	103 (33.2%)	0.373
• Rural	46 (61.3%)	29 (38.7%)	
**Occupation**			
• Physicians	144 (73.1%)	53 (26.9%)	**0.002**
• Nurse	109 (58.0%)	79 (42.0%)	
**Qualification**			
• Bachelor’s degree/Diploma in nursing	154 (60.9%)	99 (39.1%)	**0.006**
• Master’s and/or Doctoral degree	99 (75.0%)	33 (25.0%)	
**Previously studied FGM/C in curriculum**			
• Yes	139 (71.3%)	56 (28.7%)	**0.020**
• No/Don’t remember	114 (60.0%)	76 (40.0%)	
**Previously received training about FGM/C**			
• Yes	91 (77.8%)	26 (22.2%)	**0.001**
• No/Don’t remember	162 (60.4%)	106 (39.6%)	
**Attitude to FGM/C**	34.64 ± 3.86	29.75 ± 6.44	**< 0.001**
**Attitude to professional practice/medicalization of FGM/C**	10.79 ± 1.26	9.11 ± 1.79	**< 0.001**
**Correctly defined FGM/C**			
• Yes	247 (66.2%)	126 (33.8%)	0.244
• No/Don’t know	6 (50.0%)	6 (50.0%)	
**Negative consequences of FGM/C on women’s health**			
• Yes	232 (71.2%)	94 (28.8%)	**< 0.001**
• No/Don’t know	21 (35.6%)	38 (64.4%)	
**Is there is a law prohibiting the practice in Egypt**			
• Yes	183 (67.3%)	89 (32.7%)	0.315
• No	70 (61.9%)	43 (38.1%)	
**FGM/C practice is common in area where HCP grew up**			
• Yes	145 (56.9%)	110 (43.1%)	**< 0.001**
• No/Don’t know	108 (83.1%)	22 (16.9%)	
**FGM/C practice is common in HCP family/household**			
• Yes	119 (55.6%)	95 (44.4%)	**< 0.001**
• No	134 (78.4%)	37 (21.6%)	
**Previously examined a girl who underwent FGM/C**			
• Yes	229 (67.0%)	113 (33.0%)	0.147
• No	24 (55.8%)	19 (44.2%)	
**Previously saw a girl with FGM/C complications**			
• Yes	180 (70.9%)	74 (29.1%)	**0.003**
• No	73 (55.7%)	58 (44.3%)	
**Intention to have FGM/C performed on their future daughter**			
• Yes	7 (16.3%)	36 (83.7%)	**< 0.001**
• No/Don’t know	246 (71.9%)	96 (28.1%)	
**Received advice not to perform FGM/C**			
• Yes	136 (68.3%)	63 (31.7%)	0.261
• No	117 (62.9%)	69 (37.1%)	
**Ever carried out FGM/C**			
• Yes	29 (56.9%)	22 (43.1%)	0.153
• No	224 (67.1%)	110 (32.9%)	

Having a Master’s or doctoral degree, previous study of FGM/C in the academic curriculum and receiving training about FGM/C were significantly associated with support for the professional practice of FGM/C or not being clearly against FGM/C (*P* < 0.05). HCPs who supported the decision for FGM/C or were not clearly against FGM/C had significantly lower mean values for their attitude against FGM/C, both in general terms and as a professional practice, compared to those who did not support the practice.

Significantly higher proportions of support for FGM/C or not being clearly against FGM/C were reported by HCPs who were raised in areas and families where FGM/C was prevalent and by those who stated a future intention to have FGM/C performed on their daughters compared to those who did not (*P* < 0.05). A significantly lower proportion of HCPs who had previously seen a girl with FGM/C complications supported FGM/C (29.1%) compared to those who had not (44.3%) (*p* = 0.003).

Table 4 displays the adjusted predictors of support for FGM/C or not being clearly against FGM/C among HCPs in Upper Egypt. In the adjusted model, increasing age of HCPs was a significant predictor of support for FGM/C or not being clearly against FGM/C (AOR = 1.03, 95%CI: 1.01–1.06). In addition, low scores of HCPs on attitude against the professional practice of FGM/C predicted support for FGM/C or not being clearly against FGM/C (AOR = 0.610, 95%CI: 0.49–0.75). HCPs who reported FGM/C prevalence in the area they were raised had significantly higher odds ratio of supporting FGM/C or not being clearly against FGM/C compared to those who did not (AOR = 2.410, 95%CI: 1.22–4.75).

**Table 4:** Predictors of support for FGM/C as professional practice/not clearly against FGM among HCPs, upper Egypt

Predictors	Adjusted OR	95% CI., Upper, Lower	*P*-Value
**Age in years**	1.03	1.01–1.06	0.013
**Sex (Male)**		Reference group	
Female	1.553	0.74–3.25	0.243
**Qualification (Master’s/Doctoral degree)**		Reference group	
Bachelor’s degree/Diploma in nursing	1.800	0.89–3.62	0.099
**Job type (Physicians)**		Reference group	
Nurses	0.647	0.32–1.29	0.219
**Previously studied FGM/C in academic curriculum (Yes)**		Reference group	
No	1.003	0.59–1.70	0.990
**Previous received any FGM/C training (Yes)**		Reference group	
No	1.790	0.97–3.32	0.064
**General attitude towards FGM/C Score**	0.976	0.91–1.05	0.518
**Attitude towards professional practice of FGM/C Score**	**0.610**	**0.49–0.75**	**< 0.001**
**Awareness of negative consequences of FGM/C (Yes)**		Reference group	
No	1.398	0.62–3.15	0.419
**Predominance of FGM/C in area HCP grew up (No)**		Reference group	
Yes	**2.410**	**1.22–4.75**	**0.011**
**Predominance of FGM/C in HCP family/household (No)**		Reference group	
Yes	1.038	0.52–2.05	0.914
**Previously saw a girl with FGM/C complications (Yes)**		Reference group	
No	1.096	0.63–1.91	0.747
**Intention to have FGM/C performed on their future daughters (No)**		Reference group	
Yes	2.313	0.77–6.93	0.134

Adjusted logistic regression analysis was applied

## Discussion

An increasingly larger share of FGM/C is being performed by HCPs, especially in Egypt. HCPs justify the medicalization of this practice as a response to the families’ requests to perform FGM/C. The practice may also offer them financial gain or be justified on the basis of cultural and religious beliefs, or believing that the professional performance of FGM/C would decrease the risk of harm for girls or women who might otherwise rely on a traditional practitioner [18].

FGM/C cases are commonly presented in clinics of paediatricians and gynaecologists, who have the closest contact with this health issue [24]. The current study aimed to identify the prevalence of support for medicalized FGM/C among HCPs working in the specialty of gynaecology and obstetrics in Upper Egypt.

The majority of the Egyptian HCPs studied, both physicians and nurses, correctly defined the practice of FGM/C (almost 97%). Concurrently, a large proportion reported that the practice has negative consequences for girls’ health. This could be explained by the predominance of the practice in Egypt, the recruitment of HCPs from the specialty of gynaecology in the current study and almost half of the HCPs having studied FGM/C in their undergraduate curriculum.

According to the findings of a previous study in Nigeria and Sudan, despite FGM/C being widely practised in these countries and the majority of care professionals being aware of FGM/C, a limited proportion of HCPs correctly identified FGM/C types [26].

The rise of migration from countries with a high prevalence of FGM/C to developed countries that have low FGM/C prevalence rates has led to a rise in the number of women and girls who undergo FGM/C in the developed world [27]. This rise increases the probability of clinicians being exposed to women who have undergone FGM/C. However, low levels of awareness about FGM/C types have also been reported in Flemish, Australian and Spanish HCPs [20, 22, 28].

HCPs in the field of gynaecology are considered front-line staff who are the most likely to be required to assist and support women’s health. The lack of ability to correctly identify the various types of FGM/C performed will limit their professional ability to provide the most appropriate care for women who seek medical attention.

Despite the Egyptian government’s efforts to counter the practice of FGM/C and the existence of an Egyptian law that criminalizes the performance of FGM/C, about 35% of the Egyptian HCPs studied supported the decision for FGM/C or were not clearly against FGM/C.

In the adjusted logistic regression model, older age of HCPs significantly predicted their support for the professional practice of FGM/C or not being clearly against FGM/C (AOR = 1.03, 95 CI%:1.01–1.06). This may indicate lower support for FGM/C among the younger generation and consequently more understanding of FGM/C as a violation of the human rights of girls. Similarly, in previous studies, younger Spanish health professionals have been found to be more motivated to educate, address and counsel clients to improve care and prevent FGM/C [28].

Adjusted regression analysis revealed that the sex of the HCP in the current study had no significant relation to support for the professional practice of FGM/C or not being clearly against FGM/C given the scenario of being consulted by a girl’s mother (AOR = 1.553, 95 CI%:0.74–3.25). Similarly, in previous studies, no significant differences were detected between males and females among Egyptian physicians and Spanish health professionals in their attitude towards FGM/C [28, 29].

Attitudes give people a sense of identity and belonging, they may affect cognition, predict behaviour and guide their choices and actions [30]. HCPs can play an essential role in communicating with patients and providing preventive messages that may contribute to the abandonment of FGM/C. HCP beliefs are key in ensuring this takes place [31]. The impact of HCP attitudes on their practice was evident in the current study, where HCPs with a negative attitude towards medicalization and the professional practice of FGM/C significantly showed low support for FGM/C as a professional practice (AOR = 0.610, 95 CI%: 0.49–0.75).

Childhood experiences in their family and social life may affect HCPs’ perceptions of gender roles and their professional choices [32]. In accordance with this, the present study found that being raised in an area with a high prevalence of FGM/C positively affected HCPs’ support for the professional practice of FGM/C (AOR = 2.410, 95 CI%: 1.22–4.75).

The current study provides a valuable overview of the beliefs and professional choices of Egyptian HCPs regarding FGM/C, as well as revealing the underlying factors guiding their behaviour. However, this study has some limitations. The results only concern HCPs in Upper Egypt in the specialty of gynaecology and obstetrics. There is a need for a more comprehensive study that covers various regions in Egypt and incorporates other essential specialties that mothers may consult in relation to FGM/C, such as paediatricians and general and plastic surgeons.

Moreover, the researchers used a purposive sampling technique to recruit HCPs. This was due to the nature of the latter’s work in the clinical field, having limited free time from their clinical work in the hospitals, especially physicians. The applicability of such non-probability sampling might make it a challenge to generalize the study findings.

## Conclusions

Despite the majority of the HCPs studied being aware of the negative consequences of FGM/C and the existence of a law prohibiting FGM/C in Egypt, there is still a substantial proportion of Egyptian HCPs who support FGM/C or are not clearly against FGM/C, when given the scenario of being consulted about FGM/C. Egyptian HCPs who are older, who have been raised in areas where FGM/C is prevalent and who have a positive attitude towards the professional practice of FGM/C are more supportive or not clearly against FGM/C.

### Recommendations

There is a need to direct educational programmes that challenge FGM/C towards Egyptian HCPs, targeting the cultural values that lead them to support the professional practice of FGM/C. More intensified efforts should be directed at older HCPs, those who have a positive attitude towards FGM/C medicalization and those who were raised in areas with a predominance of FGM/C.

## Supplementary Information

Below is the link to the electronic supplementary material.

Supplementary Material 1

## Data Availability

The data sets generated and analyzed during the current study are available from the corresponding author on reasonable request.

## Abbreviations

FGM/C: Female Genital Mutilation/Cutting
HCPs: Healthcare Providers
WHO: World Health Organization
EFHS: Egyptian Family Health Survey
