# Supplementary material for: Supporting the decision for female genital mutilation/cutting and its predictors among healthcare providers in Upper Egypt
Source: BMC Health Serv Res. 2026 Feb 4;26:313. doi: 10.1186/s12913-026-14028-w (PMC12937513; doi:10.1186/s12913-026-14028-w)
Supplement: Supplementary file 1 — Supplementary Material 1 [file 12913_2026_14028_MOESM1_ESM.docx]

**Supporting the decision of female genital mutilation/ cutting practice and its predictors among healthcare providers in Upper Egypt**

**An English version**

|  | **Personal data** |  | | |
| --- | --- | --- | --- | --- |
|  | Serial number | ……………………………………………………… | | |
|  | Age ( in years) | ……………………………………………………… | | |
|  | Gender | 1. Male 2. female | | |
|  | Residence at birth and growing up | 1. Urban 2. Rural | | |
|  | Current residence | 1. Urban 2. Rural | | |
|  | Education | 1. Nursing secondary education 2. Diplomat in nursing 3. Bachelor degree in nursing 4. Bachelor degree in medicine and surgery 5. Master degree 6. Doctorate degree | | |
|  | Name Work place (institution) | ……………………………………………………… | | |
|  | Professional degree | 1. Nurse 2. Physicians | | |
|  | **Previous heard / received training about FGM/C** |  | | |
|  | Have received training after your employment about FGM/C | 1. Yes 2. No | | |
|  | Have you heard about FGM/C during your medical/ nursing educational years | 1. Yes 2. No | | |
|  | **Knowledge and Attitude towards FGM/C** |  | | |
|  | Could you describe briefly the meaning of FGM/C practice? (You can choose more than one answer) | 1. Partial or total removal of the clitoral glans 2. Partial or total removal of the clitoral glans and the labia minora, with or without removal of the labia majora 3. Narrowing of the vaginal opening with the creation of a covering seal. 4. All other harmful procedures to the female genitalia for non-medical purposes | | |
|  | Do you know the prevalence rate of FGM/C in Egypt? | 1. ……………………………………………………… 2. Do not know | | |
|  | Does female circumcision have negative consequences for a woman's current or future health? | 1. Yes 2. No 3. Do not know | | |
|  | If yes, please report what is/ are consequence/s on health of FGM/C (You can choose more than one answer) | 1. Transmission of infectious diseases 2. Bleeding 3. Health problems 4. Scar formation 5. Difficulty in pregnancy 6. Difficulty during delivery 7. Reduction of sexual feelings 8. Difficult penetration during sex. 9. Others ,please mention …………………………………….. | | |
|  | **Attitude towards FGM/C** |  | | |
|  | I will mention some statements on FGM/C could you respond to them with agree, neutral or disagree from your point of view | Agree  (1) | Neutral  (2) | Disagree  (3) |
|  | It is a mandatory religious practice | Agree  (1) | Neutral  (2) | Disagree  (3) |
|  | It is a deeply rooted good cultural practice in Egypt | Agree  (1) | Neutral  (2) | Disagree  (3) |
|  | It reduces sexual feelings | Agree  (1) | Neutral  (2) | Disagree  (3) |
|  | It is a good practice | Agree  (1) | Neutral  (2) | Disagree  (3) |
|  | It is a practice for girls’ personal hygienic | Agree  (1) | Neutral  (2) | Disagree  (3) |
|  | It is a rite of passage for girls into womanhood | Agree  (1) | Neutral  (2) | Disagree  (3) |
|  | Necessary for the marriageability of girls | Agree  (1) | Neutral  (2) | Disagree  (3) |
|  | It helps to maintain girls’ virginity for their husband | Agree  (1) | Neutral  (2) | Disagree  (3) |
|  | It reduces the rate of prostitution | Agree  (1) | Neutral  (2) | Disagree  (3) |
|  | Girls that have not undergone FGM/C should be discriminated | Agree  (1) | Neutral  (2) | Disagree  (3) |
|  | It does not violate human rights/ girls’ dignity | Agree  (1) | Neutral  (2) | Disagree  (3) |
|  | All forms of genital cutting should not be allowed (e.g., illegal). | Agree  (3) | Neutral  (2) | Disagree  (1) |
|  | **Attitudes of healthcare providers towards professionals practicing FGM/C** |  | | |
|  | I will mention some questions on FGM/C could you respond to them with yes or no from your point of view |  | | |
|  | Do you think that the practice of FGM/C should continue? | 1. Yes 2. No | | |
|  | Do you think that the practice of FGM/C can ever be eliminated in The Egypt? | 1. No 2. Yes | | |
|  | Do you think HCP workers have a role to play in eliminating FGM/C? | 1. No 2. Yes | | |
|  | Do you think of ‘medicalizing’ FGM/C make the practice safer? | 1. Yes 2. No | | |
|  | Do you think medicalizing FGM/C is a good way of encouraging FGM/C? | 1. Yes 2. No | | |
|  | Do you think of ‘medicalizing’ FGM/C should be stopped at all levels? | 1. No 2. Yes | | |
|  | **Practice FGM/C** |  | | |
|  | Is FGM/C common in the area where you grew up? | 1. Yes 2. No | | |
|  | Is FGM/C practiced in your family/household? | 1. Yes 2. No | | |
|  | As a healthcare provider, have you ever performed female genital mutilation/ cutting on a girl? | 1. Yes 2. No | | |
|  | As a health care provider, have you  ever carried out FGM/C on a girl? | 1. Yes 2. No | | |
|  | In your work, have you ever examined/seen a girl with FGM/C? | 1. Yes 2. No | | |
|  | In your work, have you seen a girl with complications after FGM/C? | 1. Yes 2. No | | |
|  | If you have a daughter, do you intend to circumcise her in the future? | 1. Yes 2. No | | |
|  | **Supporting FGM/C practice** |  | | |
|  | If a women come with her daughter/ relative and asked you to perform FGM/C practice to the girl, your response will be: | 1. perform FGM/C 2. refer her to another physician to perform FGM/C 3. examine the girl and determine if FGM/C is needed 4. Educate the mother not to perform the practice 5. I will not perform FGM/C practice 6. Not perform the practice and educate the mother 7. Others (mention:……………………) | | |
|  | Is there a legal text prohibit/ criminalize FGM/C practice in the Egyptian law? | 1. Yes 2) no | | |
|  | Do you think it is a good idea for men to be concerned on the debate on FGM/C? | 1. Yes   No | | |

**Thanks for your time**
